# London Smoke

**Published:** 1898-05-28

**Authors:** 


The Hospital

A Journal of

The Medical Sciences and Hospital Administration.

Vol. XXIV.—No. 609.] Edited by Sir Henry Burdett, K.C.B., and Solomon C. Smith, M.D., M.R.C.P. [May 28, 1898.

### LONDON SMOKE.

The parliamentary activity of Mr. Labouchere—whose mental grasp may be so far said to resemble the trunk of an elephant that nothing is too strong to be rent asunder by it, and nothing is too minute for its apprehension—has lately been occupied upon the question of what he not inappropriately called the "Smoke Nuisance at Westminster." The honourable gentleman asked the First Commissioner of Works whether he had noticed that a high chimney in close vicinity to the Palace of Westminster emits all day clouds of black smoke, and whether he would call the attention of the local authorities to the nuisance, with a view to its discontinuance. Mr. Akers-Douglas had noticed the occurrence complained of, and thought it might probably be attributed to the impossibility of obtaining Welsh coal during the strike; but he added that he had no authority in the matter, and that any representation with regard to it should be addressed to the London County Council. This part of his reply was received by the House of Commons with irreverent laughter, and the right honourable gentleman did not notice Mr. Labouchere's further suggestion that he should himself, as representing householders in the neighbourhood, make application to the august body to which he had referred.

Although (as a bit of Parliamentary repartee) the answer given to Mr. Labouchere was very smart, the County Council has really but little to do with the matter, and it is somewhat amusing to see writers in the papers, even writers in the *Lancet*, running astray in a matter like this, and all following their leader. As a fact, the control of the smoke nuisance, as of other nuisances, lies with the local sanitary authorities; in other words, with the vestries; and the County Council can but interfere (with the vestries, not with the smoke) in case of their default.

In regard to the question of London smoke, it must be borne in mind that the contributions to the nuisance which are furnished by "high" chimneys, although more conspicuous than others to the casual observer, have till lately been in the aggregate comparatively unimportant. The whole subject has been threshed out by a series of parliamentary committees, and the Acts of 1845 and 1847 have been followed by much further legislation directed to the control of the smoke of manufacturing and other industries. The fact remains that these industries have not been responsible for more than a small part of the nuisance, and that the innumerable open fire grates of the metropolis, with their estimated consumption of some fifty thousand tons of coal daily, have been and still are the main offenders in the matter, and are offenders that it would be scarcely possible to bring under any kind of regulation. Defective fire-grates can only be eliminated gradually, by the aid of a heightened perception of the injury done by smoke, and by the introduction of gas and electricity as heating agents for many ordinary purposes.

It must be obvious, however, to anyone who keeps his eyes open that a considerable change has crept over the aspect of London during the last few years. That the general smokiness of the atmosphere has diminished, and that the general murkiness which each morning creeps over the town, coincidentally with the cooking of some millions of breakfasts, is less than it was some six or eight years ago, is generally recognised.

At the same time, however, that this amelioration of the nuisance from domestic smoke has been taking place, no one can doubt that there has been a very considerable increase, in the West-end at any rate, of that arising from tall chimneys. No doubt this is in part the result of the supervision of the chimneys having been removed from the police and placed in the hands of the sanitary authorities, hands that have not yet earned for themselves the same reputation for stern impartiality as was held by their predecessors. To a large extent, however, the increase of "dense black" smoke is due to the great multiplication of furnaces which has taken place during recent years. Many of the great buildings, which are such a striking feature in modern London, are provided with engines and with furnaces, which consume almost as much coal as if they belonged to manufacturing establishments. Hotels and theatres and restaurants have their staffs of engineers and stokers, just as if they were mills, and when the slightest pressure comes upon them, or when, as no doubt has lately been the case to some extent, they have not been able to get the particular sort of coal which their furnaces were more especially devised to burn, dense smoke has been poured out in abundance. Added to all this is the fact that the spread of electric lighting has caused what is to all intents and purposes a great manufacturing industry, consuming an enormous amount of coal, to be planted in the very midst of many residential centres. Undoubtedly, the electric light companies are often terrible sinners as to smoke, and whole districts are now and again smothered by them in dense black clouds, such as no Lancashire furnace-man would think of producing. It is easy to say that all this is preventable. So it is; but what one has to bear in mind is that, in face of a growing trade and sudden calls for an enormous production of electricity, and in face, at the same time, of the great expense involved in the provision of sites on which to enlarge electric lighting stations, the lighting companies are on the horns of a dilemma, and find that it is cheaper to force their furnaces and produce smoke than it is to extend their premises. The same in regard to the furnaces in other buildings. The use of, and the demand for "power" is everywhere extending; furnaces are everywhere being made to work to their utmost, and it is cheaper to make smoke—and even to pay fines—than to enlarge apparatus. There is every year a greater and greater temptation to disregard the law until it is stringently enforced. The moral of this is obvious, namely, an increased vigour in the application of the law. So far as prosecution for the production of black smoke from furnaces is concerned, the sanitary authorities are furnished with the most absolute powers, if they would but exert them. It is not necessary for them to prove a nuisance; all they have to do is to show that the smoke is black, and that it is caused by a "manufacturing or trade process." The difficulty lies in the Courts; for, unless the magistrates can be induced to impose effective penalties, and to repeat them for each offence, the smokers will continue to find it the cheaper course to pay rather than to reform.

